# miR-155 is dispensable in monosodium urate-induced gouty inflammation in mice

**DOI:** 10.1186/s13075-018-1550-y

**Published:** 2018-07-11

**Authors:** Qibin Yang, Quanbo Zhang, Yufeng Qing, Li Zhou, Qingsheng Mi, Jingguo Zhou

**Affiliations:** 1 Department of Rheumatology and Immunology, Affiliated Hospital of North Sichuan Medical College, Sichuan Province, Nanchong, 637000 China; 2 Department of Gerontology, Affiliated Hospital of North Sichuan Medical College, Sichuan Province, Nanchong, 637000 China; 3 Henry Ford Immunology Program, Henry Ford Health System, 1 Ford Place, Detroit, MI 48202 USA; 4 Department of Dermatology, Henry Ford Health System, 1 Ford Place, Detroit, MI 48202 USA; 5 Department of Internal Medicine, Henry Ford Health System, 1 Ford Place, Detroit, MI 48202 USA; 6 Department of Rheumatology and Immunology, The First Affiliated Hospital of Chengdu Medical College, Sichuan Province, Chengdu, 610000 China

**Keywords:** MiR-155, MSU, Gout, Inflammation

## Abstract

**Background:**

The findings of a previous study by Jin et al. have shown that microRNA (miR)-155 was upregulated in patients with acute gouty arthritis and enhanced the proinflammatory cytokines. There is no direct evidence to support that miR-155 is indeed involved in monosodium urate (MSU)-induced inflammatory responses *in vivo*. The aim of this study was to investigate the role of miR-155 knock-out (KO) or knock-in (KI) mice in MSU-induced animal models to mimic acute gout.

**Methods:**

MiR-155 expression in cultured bone marrow-derived macrophages (BMDMs) from miR-155 KO, miR-155 KI, and wild-type (WT) mice treated with MSU crystals *in vitro* was detected by real-time quantitative polymerase chain reaction (qPCR). MiR-155 KO and WT mice were used to induce an acute gouty inflammatory response with MSU crystals including models of foot pad inflammation, ankle arthritis, air pouch inflammation, and peritonitis. Furthermore, the proinflammatory interleukin (IL)-1β levels in lavage fluids from air pouch and peritoneal cavity models were measured by enzyme-linked immunosorbent assay (ELISA), and tumor necrosis factor (TNF)-α production from BMDMs of miR-155 KI mice treated with MSU were measured by flow cytometry.

**Results:**

MiR-155 expression was quickly upregulated in BMDMs from WT mice following MSU treatment *in vitro*. In comparison with WT mice *in vivo*, the swelling index of miR-155 KO mice showed no significant difference in the murine foot pad and ankle arthritis models for the indicated different time points. There were similar changes in total cell numbers of lavage fluids in the air pouch and peritoneal cavity models between miR-155 KO and WT mice following MSU crystal injection. Moreover, the IL-1β levels of lavage fluids in the air pouch and peritonitis models from miR-155 KO mice were almost the same as those from WT mice. TNF-α levels were comparable from BMDMs treated with MSU crystals *in vitro* between miR-155 KI mice and WT mice.

**Conclusions:**

MiR-155 is dispensable in MSU-induced gouty inflammation in mice. Deletion of miR-155 might not be an effective therapeutic approach to relieve the inflammation in acute gout.

## Background

Gout is one of the most common forms of inflammatory arthritis disorder and is caused by deposition of monosodium urate (MSU) crystals in and around the joints [1]. Accumulated studies indicate that at least two pathways, the Toll-like receptor (TLR)/nuclear factor (NF)-κB signaling pathway and the NLR family pyrin domain containing 3 (NLRP3) pathway, are involved in MSU crystal-induced inflammatory cytokine release from monocytes/macrophages [2–4]. However, the molecular mechanisms of gouty inflammation are still not entirely elucidated.

MicroRNAs (miRNAs) are an abundant class of small, evolutionary conserved, non-coding RNAs acting as post-transcriptional regulators. Our previous data have reported that miRNAs regulate the immune cell development and function, and control autoimmune disease development [5, 6]. Aberrant miRNA expression has been observed in a number of diseases. Recent studies suggest that miRNAs are potentially involved in the development of human acute gouty arthritis, including miR-155 [7] and miR-146a [8]. Dalbeth et al. [8] found that miR-146a expression increased in both human monocytic THP-1 cells and human peripheral blood mononuclear cells (PBMCs) using MSU crystal stimulation in vitro, but the expression of other miRNAs (including miR-155, miR-146b, miR-9, and miR-21) did not, implicating interleukin (IL)-1β regulation. Interestingly, higher levels of miR-146a were expressed in PBMCs from intercritical gout patients when compared with both control groups (normouricemic control participants/hyperuricemic control participants) and the acute gout group. Additionally, miR-146a expression was reduced during the acute flare compared with the intercritical period in the paired samples as well as in the urate peritonitis model. Those findings reflect the more complex multicellular *in vivo* response to MSU crystals. Numerous studies have reported that miR-155 could negatively regulate the TLR/NF-κB signaling pathway by targeting different kinds of genes [9], and that miR-155 deficiency reduced inflammatory responses in the colitis mouse model [10]. MiR-155 expression, which is closely related to disease activity, is upregulated in the PBMCs and synovial membrane of rheumatoid arthritis [11, 12]. Jin et al. [7] showed that miR-155 was upregulated in synovial fluid mononuclear cells (SFMCs) from patients with acute gouty arthritis and MSU crystals strongly induced miR-155 expression in PBMCs *in vitro*. Furthermore, the increased expression of miR-155 in SFMCs led to downregulation of the SH2 domain-containing inositol 5′-phosphatase 1 (SHIP1), which activates the Akt/NF-kB pathway and enhances the production of proinflammatory cytokines, including IL-1β. Notably, miR-155 levels in PBMCs from gout patients were comparable with healthy individuals. These paradoxical data suggest that the same miRNA may play different roles in different tissues/organs and conditions. However, there is still a lack of direct evidence to further determine whether miR-155 is indeed involved in MSU-induced inflammation *in vivo*.

In the present study, we took advantage of miR-155 knock-out (KO) mice to investigate the role of miR-155 in MSU-induced gout *in vivo*. Four MSU-induced gout mouse models, including foot pad inflammation, ankle arthritis, peritonitis, and air pouch inflammation, were used. The proinflammatory cytokine IL-1β levels in lavage fluids from the peritoneal cavity and air pouch models and tumor necrosis factor (TNF)-α levels from bone marrow-derived macrophages (BMDMs) of miR-155 knock-in (KI) mice treated with MSU crystals were measured.

## Methods

### Animals

MiR-155^−/−^C57BL/6 knock-out (miR-155 KO) mice, Csf1r^+^155Tg/Tg knock-in (miR-155 KI) mice, and Csf1r^−^155Tg/Tg wild-type (WT) mice were kindly provided by the CBR Institute for Biomedical Research, Harvard Medical School, Boston, USA. C57BL/6 as WT mice were purchased from the Jackson Laboratory. Colony-stimulating factor 1 receptor (Csf1r), also known as macrophage colony-stimulating factor (M-CSF) receptor, controls the production, differentiation and function of macrophages. As we know, macrophages play crucial roles (such as phagocytosis and trigging the inflammatory response) in acute gout including in the initial phase, in development, and in remission. The mice were housed at 24 ± 2 °C under 12-h light/12-h dark cycles in a pathogen-free facility; 8- to 10-week-old males were used to perform the experiments. The handling of mice and experimental procedures in this study were performed in accordance with the requirements of the Institutional Animal Care and Use Committee of Henry Ford Health System.

### Preparation of MSU crystals

Briefly, 1.0 g uric acid (Sigma-Aldrich) was dissolved in 200 ml boiling distilled water containing 6.0 ml 1 M NaOH. After adjusting the pH of the solution to 7.2 with HCl, crystals that formed were sterilized by heating at 180 °C for 2 h [13]. The solution was gradually cooled by stirring at room temperature and stored overnight at 4 °C. The precipitate was filtered from the solution, dried under low heat, and suspended in phosphate-buffered saline (PBS) at a concentration of 50 mg/ml. All reagents were prepared under pyrogen-free conditions.

### BMDM culture and MSU stimulation

Bone marrow cells obtained from the femoral bones of C57BL/6(WT), miR-155 KO, Csf1r^+^155Tg/Tg (miR-155 KI), and Csf1r^−^155Tg/Tg (WT) mice, respectively, were cultured in RPMI 1640 (Gibco) supplemented with 10% fetal bovine serum (FBS), penicillin (100 units/ml), and streptomycin (100 μg/ml). To induce the proliferation and differentiation of myeloid progenitors to macrophages, the medium was supplemented with 30 ng/ml M-CSF (#0914245, Peprotech). The cells were washed and received fresh medium with M-CSF every 2–3 days. After 7 days the cells were harvested with 0.25% trypsin. Dead cells were first gated out by propidium iodide (PI) staining. The phenotypic validation of BMDMs was performed by flow cytometry with staining using fluorescein isothiocyanate (FITC)-conjugated anti-CD11b and phycoerythrin (PE)-conjugated anti-F4/80 antibodies (both diluted 1:200). The BMDMs are double-positive for CD11b and F4/80. According to the experimental protocol, the MSU crystal suspension (MSU 2.5 mg/ml concentration) was added to the incubated BMDMs (MSU 100 μg/ml final concentration) for 4 or 8 h and the ratio of TNF-α production from BMDMs treated with MSU for 2 or 4 h were measured.

### Gout model

Mice were placed under anesthesia (150:10 mg/kg ketamine:xylazine injected intraperitoneally) and were injected with MSU crystals into the right foot pad (1 mg in 40 μl PBS) or ankle joint (0.5 mg in 20 μl PBS). The same volume of sterile saline was injected into the left foot pad or ankle joint at the same time to serve as the control. Inflammation parameters were evaluated following MSU crystal injection at different time points (6, 24, and 48 h). Paw swelling and the size of ankle joints were measured with an electronic caliper at the indicated time points [14–16]. For an air pouch model, 5 ml sterile air was first injected subcutaneously into the back of mice to form an air pouch, and then 3 ml sterile air was injected to the air pouch at day 3 and day 5. At day 7, MSU suspension (3 mg in 1 ml PBS) was injected into the air pouch [17]. For MSU-induced peritonitis, MSU crystals (3 mg in 0.5 ml PBS) were injected into the peritoneal cavity as part of the intraperitoneal gouty model [7]. The total number of air pouch and peritoneal cavity exudate cells were harvested after 4 or 8 h and counted by a hemocytometer.

### Real-time quantitative polymerase chain reaction

The cultured BMDMs were harvested after 7 days. Total RNA of BMDMs was extracted using Trizol reagent (Invitrogen, USA) and reverse-transcribed into cDNA using reverse transcription reagents (Invitrogen, USA) according to the manufacturer’s protocols. Real-time quantitative polymerase chain reaction (qPCR) was performed using the ABI Prism 7900HT Detection System (Applied Biosystems, USA) with Power SYBR Green PCR Master Mix (Applied Biosystems, USA). The gene primers sequences were synthesized by eurofins genomics (Louisville, USA) as follows. TNF-α: forward 5’-ACAAAGGTGCCGCTAACCACATGT-3′, reverse 5’-ATGCTGCTGTTTCAGTCGAAGGCA-3′; IL-1β: forward 5’-GGGCCTCAAAGGAAAGAATC-3′, reverse 5’-CTCTGCTTGTGAGGTGCTGA-3′; β-actin: forward 5’-CAACGAGCGGTTCCGATG-3′, reverse 5’-GCCACAGGATTCCATACCCA-3′. The β-actin as an internal control was used to normalize the gene expression. Gene expression was analyzed using the 2^*–ΔΔCT*^ method.

The expression of miR-155 in BMDMs was measured using Taqman MicroRNA Assays (Applied biosystems, Foster City, CA, USA) according to the manufacturer’s protocols. The Taqman MicroRNA Assay for U6 snRNA was used to normalize the relative abundance of miRNAs.

### Enzyme-linked immunosorbent assay (ELISA)

IL-1β protein levels in lavage fluids of the air pouch and peritoneal cavity models were measured by ELISA using kits from eBioscience (cat. no. 88–7013-88; San Diego, CA, USA) following the manufacturer’s instructions. The 96-well microplates were read using a VICTOR X3 plate reader.

### Statistical analysis

Data were analyzed with GraphPad Prism 5. Differences between experimental groups were tested using the unpaired *t* test. Data are expressed as mean ± SEM. *P* < 0.05 was considered to denote statistical significance.

## Results and discussion

**Fig. 1:**
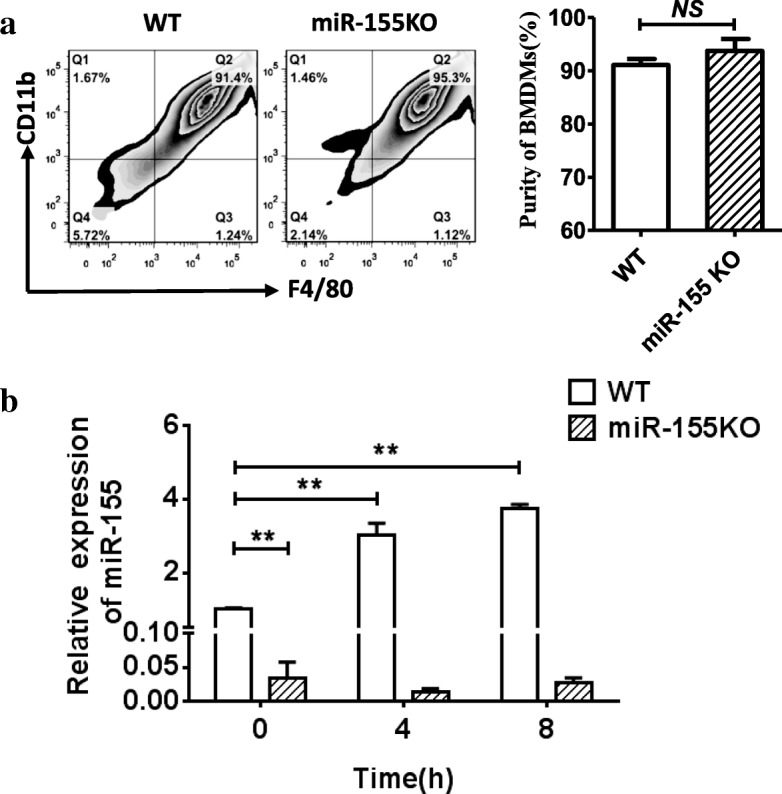
MiR-155 is upregulated in bone marrow-derived macrophages. **a** Bone marrow-derived macrophages (BMDMs) from miR-155 knock-out (KO) and wild-type (WT) mice were cultured after 7 days and the purity of BMDMs analyzed by flow cytometry. The BMDMs were double-positive for CD11b and F4/80. **b** BMDMs from miR-155KO and WT mice were treated with MSU for 4 and 8 h, and miR-155 expression was detected by Taqman real-time qPCR. Results are representative of three independent experiments; *n* = 3–5 mice per group. **P < 0.01. *NS* not significant

In the present study, based on the findings from Jin et al. [7] who found that aberrant miR-155 expression was involved in the pathogenesis of gout, we further investigated the role of miR-155 in MSU-mediated gout using miR-155 KO or KI mice in *in-vitro* or *in-vivo* experiments. We firstly analyzed the miR-155 expression from BMDMs of miR-155 KO and WT mice with or without MSU treatment *in vitro*. The purity of cultured BMDMs was no different between miR-155 KO and WT mice (Fig. 1a). The level of miR-155 expression in BMDMs from miR-155 KO mice was significantly lower than that from WT mice (Fig. 1b). The previously described study reported that miR-155 expression in the THP-1 cell lineage experiment *in vitro* was comparable with or without MSU crystal stimulation [8]. However, miR-155 expression could be induced by the MSU crystal stimulation in the PBMCs of healthy controls *in vitro* [7]. Our results showed that miR-155 expression was quickly and strongly upregulated in BMDMs from WT mice following MSU crystal treatment, while miR-155 expression was hardly induced in miR-155 KO mice (Fig. 1b). This is consistent with the previous report [7] of higher levels of miR-155 expression due to MSU crystal stimulation.

**Fig. 2:**
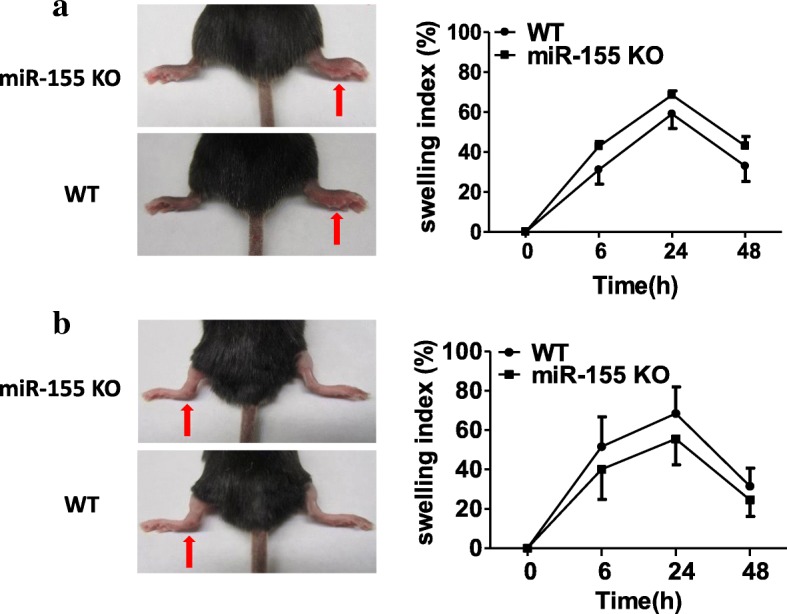
Acute gouty inflammation was induced in miR-155 knock-out (KO) and wild-type (WT) mice by MSU. **a** Swelling index of the foot pad model was measured by an electronic caliper at the different time points in miR-155 KO and WT mice treated with MSU (1 mg in 40 μl PBS). **b** Swelling index of the ankle joint model was measured by an electronic caliper at the different time points after MSU treatment (0.5 mg in 20 μl PBS). Results are representative of three independent experiments; *n* = 5 mice per group

We next tried to better understand whether miR-155 deficiency affects the inflammatory response in MSU-induced gouty inflammation *in vivo*. According to a description of murine gout models [14–16], the manifestation of inflammation such as redness and a swollen joint, and the swelling index were used to evaluate the inflammatory levels. We used MSU-induced foot pad inflammation and MSU-induced ankle arthritis models. As shown in Fig. 2, although the swelling indexes increased following MSU crystal treatment and reached a peak of swelling at 24 h, the swelling indexes were no different between miR-155 KO mice and WT mice in either the foot pad model (Fig. 2a) or the ankle joint model (Fig. 2b). These results indicate that deletion of miR-155 expression does not significantly affect the phenotype of MSU-induced gouty inflammation *in vivo*.

**Fig. 3:**
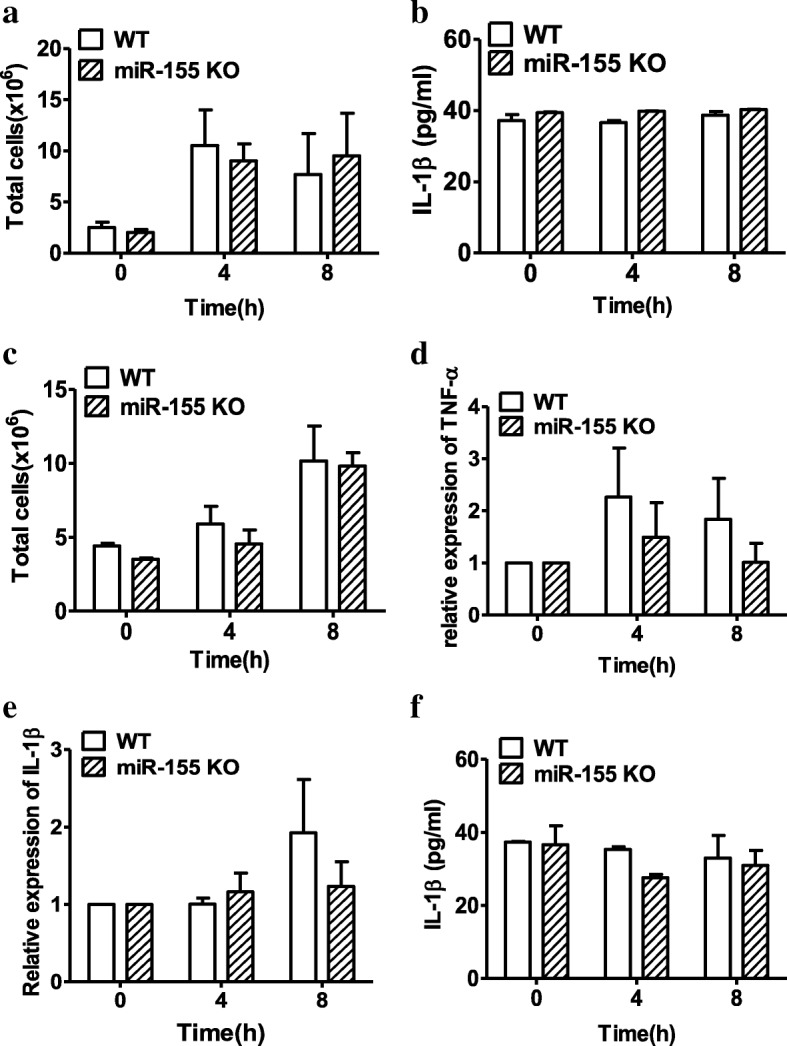
Acute gouty inflammation was induced in the peritoneal cavity and air pouch models from miR-155 knock-out (KO) and wild-type (WT) mice treated with MSU. **a, c** Total cell number in lavage fluids from the peritoneal cavity (**a**) and air pouch (**c**) models were counted by a hematocytometer. **d, e** The mRNA expression of tumor necrosis factor (TNF)-α (**d**) and interleukin (IL)-1β (**e**) were measured by real-time qPCR in the total cells from air pouch lavage fluids of miR-155 KO and WT mice treated with MSU crystals for 0, 4, or 8 h. **b, f** The cytokine IL-1β levels in lavage fluids from peritoneal cavity (**b**) and air pouch (**f**) models were measured by ELISA. Results are representative of three independent experiments; *n* = 3–5 mice per group

An acute inflammatory profile occurs in response to MSU crystals; infiltration of neutrophils and monocytes/macrophages into the peritoneum was observed after 4 h, and proinflammatory cytokines such as IL-1β, IL-6, and TNF-α were elevated within 2 h and peaked at 4 h [18]. Given that elevated levels of miR-155 are involved in MSU-induced gouty inflammation, we hypothesized that loss of miR-155 may reduce the inflammatory mediator of MSU-induced gout. To test this hypothesis, MSU crystals were injected into the peritoneal cavity of miR-155 KO and WT mice, referring to Jin et al.’s protocol [7] who used the synovial cavity, to induce an acute inflammatory response. Mice were sacrificed at 0, 4, or 8 h following MSU crystal injection. The total cell number of lavage fluids was counted in the peritonitis model, and there were similar levels of total cell numbers between miR-155 KO and WT mice regardless of a dramatically increasing tendency observed in both of these as time goes on (Fig. 3a). We also used an MSU-induced air pouch model, a model system characterized by the generation of a synovium-like lining cell layer [19]. There were also no remarkable changes in total cell numbers in lavage fluids in the air pouch (Fig. 3c) between miR-155 KO and WT mice following MSU treatment. Additionally, TNF-α (Fig. 3d) and IL-1β (Fig. 3e) mRNA expression from the air pouch lavage fluids cells were not significantly different between miR-155 KO and WT mice. Finally, we also found that the proinflammatory cytokine IL-1β levels in lavage fluids from the peritoneal cavity (Fig. 3b) and the air pouch (Fig. 3f) from miR-155 KO mice were comparable with those from WT mice. Thus, our findings indicate that the loss of miR-155 is not an effective relief strategy for MSU-induced air pouch and peritonitis *in vivo.*

**Fig. 4:**
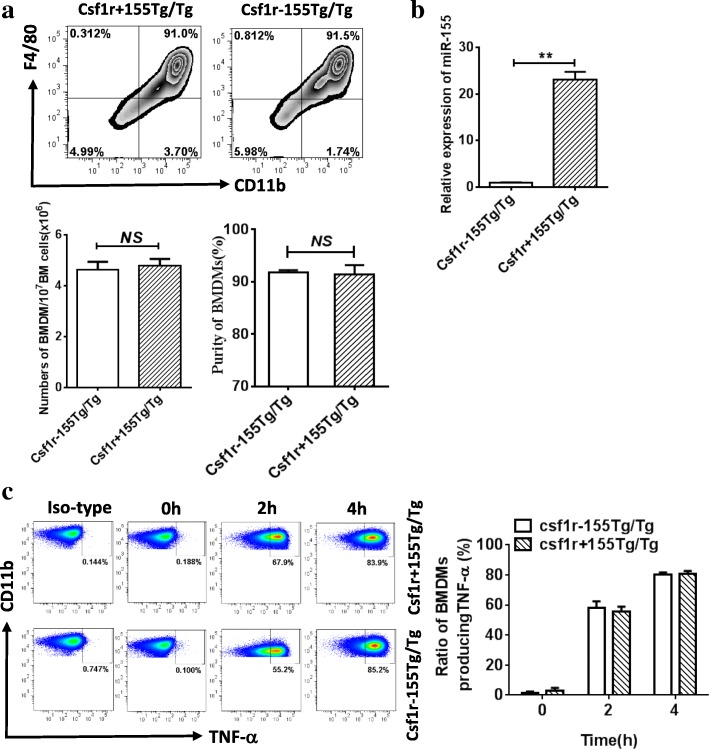
Cytokine tumor necrosis factor (TNF)-α was produced from BMDMs of Csf1r^+^155Tg/Tg (KI) and Csf1r^−^155Tg/Tg (WT) mice with MSU stimulation. **a** Bone marrow-derived macrophages (BMDMs) from miR-155 KI and WT mice were cultured for 7 days, and the purity and number of BMDMs were analyzed by flow cytometry. The BMDMs were double-positive for CD11b and F4/80. **b** MiR-155 expression was detected by Taqman real-time qPCR in BMDMs from miR-155 KI and WT mice. **c** BMDMs from miR-155KI and WT mice were treated with MSU for 0, 2, or 4 h, and the ratio of cytokine TNF-α production from BMDMs following MSU treatment was assayed by flow cytometry. Results are representative of three independent experiments; *n* = 3 mice per group. **P < 0.01. *NS* not significant

It has been found that overexpression of miR-155 promotes TNF-α and IL-1β levels in the supernatant of THP-1 cells treated with MSU *in vitro* [7]. Thus, we further assessed the effects of miR-155 overexpression on the MSU-induced inflammatory response. The BMDMs from Csf1r^+^155Tg/Tg (miR-155 KI) and Csf1r^−^155Tg/Tg (WT) mice with MSU stimulation were used in the present study. In comparison with WT mice, both purity and number of cultured BMDMs were comparable in miR-155 KI mice (Fig. 4a). We validated the miR-155 expression in BMDMs from miR-155 KI mice. As shown in Fig. 4b, a dramatically increased expression of miR-155 was observed in BMDMs from miR-155 KI mice compared with those from WT mice. The cytokine TNF-α levels from BMDMs following MSU treatment for 2 or 4 h were assayed by flow cytometry. We found that the percentage of BMDMs producing TNF-α in miR-155 KI mice was similar to that of WT mice although overexpression of miR-155 was likely to promote more TNF-α production (Fig. 4c). Our results further support that miR-155 does not directly affect MSU-induced gouty inflammation. This is in disagreement with the previous result of high miR-155 expression promoting TNF-α production [7]. This discrepancy might result from overexpression of miR-155 *in vitro or in vivo*. More research is required to untangle the confusing role of miR-155 in gouty inflammation.

Overall, our data suggest that miR-155 is dispensable in MSU-induced gouty inflammation in mice. Given that many miRNAs are regulated in monocytes/macrophages upon MSU stimulation (our unpublished data), miR-155 is more likely redundant with other regulated miRNAs during gout development.

## Conclusion

The miR-155 expression was quickly upregulated in BMDMs from WT mice with MSU-induced acute gouty inflammation *in vitro.* MiR-155 deficiency did not significantly affect the phenotype in diverse murine MSU-induced gouty inflammation *in vivo*. Therefore, deletion of miR-155 might not be an effective therapeutic approach to relieve the inflammation in acute gout.

## Abbreviations

BMDM: Bone marrow-derived macrophage
Csf1r: Colony-stimulating factor 1 receptor
ELISA: Enzyme-linked immunosorbent assay
IL: Interleukin
KI: Knock-in
KO: Knock-out
M-CSF: Macrophage colony-stimulating factor
miRNA: MicroRNA
MSU: Monosodium urate
NF: Nuclear factor
NLRP3: NLR family pyrin domain containing 3
PBMC: Peripheral blood mononuclear cell
PBS: Phosphate-buffered saline
qPCR: Quantitative polymerase chain reaction
SFMC: Synovial fluid mononuclear cell
SHIP1: SH2 domain-containing inositol 5′-phosphatase 1
TNF: tumor necrosis factor
TLR: Toll-like receptor
WT: Wild-type
