# Correction to: miR-155 is dispensable in monosodium urate-induced gouty inflammation in mice

**DOI:** 10.1186/s13075-018-1696-7

**Published:** 2018-08-14

**Authors:** Qibin Yang, Quanbo Zhang, Yufeng Qing, Li Zhou, Qingsheng Mi, Jingguo Zhou

**Affiliations:** 1 Department of Rheumatology and Immunology, Affiliated Hospital of North Sichuan Medical College, Nanchong, 637000 Sichuan Province China; 2 Department of Gerontology, Affiliated Hospital of North Sichuan Medical College, Nanchong, 637000 Sichuan Province China; 3 Henry Ford Immunology Program, Henry Ford Health System, 1 Ford Place, Detroit, MI 48202 USA; 4 Department of Dermatology, Henry Ford Health System, 1 Ford Place, Detroit, MI 48202 USA; 5 Department of Internal Medicine, Henry Ford Health System, 1 Ford Place, Detroit, MI 48202 USA; 6 Department of Rheumatology and Immunology, The First Affiliated Hospital of Chengdu Medical College, Chengdu, 610000 Sichuan Province China

## Correction

Unfortunately, after publication of this article [1], it was noticed that 2 authors were erroneously mentioned as co-first authors. Jieun Jang and Youngsook Kim are not affiliated with this paper and the note has been removed from the original article to reflect this.
